# Comparison of anterior cingulate vs. insular cortex as targets for real-time fMRI regulation during pain stimulation

**DOI:** 10.3389/fnbeh.2014.00350

**Published:** 2014-10-09

**Authors:** Kirsten Emmert, Markus Breimhorst, Thomas Bauermann, Frank Birklein, Dimitri Van De Ville, Sven Haller

**Affiliations:** ^1^Department of Radiology and Medical Informatics, University of GenevaGeneva, Switzerland; ^2^Medical Image Processing Laboratory, Institute of Bioengineering, Ecole Polytechnique Fédérale de LausanneLausanne, Switzerland; ^3^Department of Neurology, University Medical Center of the Johannes Gutenberg-University MainzMainz, Germany; ^4^Institute of Neuroradiology, University Medical Center of the Johannes Gutenberg-University MainzMainz, Germany

**Keywords:** real-time fMRI neurofeedback, realtime fMRI, pain, anterior cingulate cortex (ACC), anterior insular cortex, insular cortex

## Abstract

Real-time functional magnetic resonance imaging (rt-fMRI) neurofeedback allows learning voluntary control over specific brain areas by means of operant conditioning and has been shown to decrease pain perception. To further increase the effect of rt-fMRI neurofeedback on pain, we directly compared two different target regions of the pain network, notably the anterior insular cortex (AIC) and the anterior cingulate cortex (ACC). Participants for this prospective study were randomly assigned to two age-matched groups of 14 participants each (7 females per group) for AIC and ACC feedback. First, a functional localizer using block-design heat pain stimulation was performed to define the pain-sensitive target region within the AIC or ACC. Second, subjects were asked to down-regulate the BOLD activation in four neurofeedback runs during identical pain stimulation. Data analysis included task-related and functional connectivity analysis. At the behavioral level, pain ratings significantly decreased during feedback vs. localizer runs, but there was no difference between AIC and ACC groups. Concerning neuroimaging, ACC and AIC showed consistent involvement of the caudate nucleus for subjects that learned down-regulation (17/28) in both task-related and functional connectivity analysis. The functional connectivity toward the caudate nucleus is stronger for the ACC while the AIC is more heavily connected to the ventrolateral prefrontal cortex. Consequently, the ACC and AIC are suitable targets for real-time fMRI neurofeedback during pain perception as they both affect the caudate nucleus, although functional connectivity indicates that the direct connection seems to be stronger with the ACC. Additionally, the caudate, an important area involved in pain perception and suppression, could be a good rt-fMRI target itself. Future studies are needed to identify parameters characterizing successful regulators and to assess the effect of repeated rt-fMRI neurofeedback on pain perception.

## Introduction

Pain perception has a great impact on individual emotional health as pain is associated with anxiety (Asmundson and Katz, 2009), anger (Trost et al., 2012), fear (Leeuw et al., 2007a,b; Vlaeyen and Linton, 2012), and worry (Eccleston and Crombez, 2007; Linton, 2013). Thus, not surprisingly, chronic pain increases the risk of depression and suicide (Turk et al., 1995; Geisser et al., 2000; Bair et al., 2003; Ilgen et al., 2008; Denkinger et al., 2014). Pharmacological intervention remains the mainstay of chronic pain treatment. As most chronic pain patients are treated with a combination of pain medications and over long periods of time (Muller-Schwefe et al., 2011), cumulative drug-related side effects pose a considerable risk of adverse effects for these patients (Jouini et al., 2014), highlighting the importance of alternative and supplementary pain therapies.

One novel technique that shows potential in the treatment of chronic pain is real-time functional magnetic resonance imaging (rt-fMRI), which allows volitionally influencing activation of a targeted brain area by means of operant conditioning when being supplied with a corresponding feedback signal. This technique could be employed to reduce brain activation in pain network target areas with the aim to decrease the subjective pain perception. A pilot study showed that it is possible to regulate the anterior cingulate cortex (ACC) as a target brain region using rt-fMRI for chronic pain patients as well as healthy participants during pain perception (Decharms et al., 2005). However, according to subsequent reports of the same group, these findings could not be replicated (Decharms, 2012). In line with this observation, rt-fMRI is generally still in its early days, facing some limitations and confounds. High inter-individual differences in regulation success and small effect sizes make it difficult to assess the therapeutic use of this method. In an attempt to optimize the choice of the target region, which is a key factor of the rt-fMRI experiment, this study compares two possible target brain regions for feedback involved in pain processing in healthy subjects. The effect of the feedback on these target regions and other brain regions within the pain-responsive network will be assessed.

Acute pain perception starts with an external stimulus that activates peripheral receptors such as the vanilloid receptor (TRPV1), which is sensitive to temperatures above 43°C (Cesare and McNaughton, 1996) eliciting a depolarization of peripheral sensory neurons synapsing onto second-order dorsal horn neurons (Basbaum and Jessell, 2000) in the spinal cord. These fibers ascend to the thalamus relaying information to the somatosensory cortex, the ACC and the insular cortex (IC). Additional projection neurons from the dorsal horn to the parabrachial nucleus in the brainstem engage the ACC and the IC via the amygdala. Apart from this ascending connection, cortical pain areas such as the primary and secondary somatosensory cortex as well as the posterior insula (PIC), which are implicated in basic pain perception, are heavily interconnected (Apkarian et al., 2005). The same is true for higher-level areas involved in pain processing, including the ACC, the anterior insula (AIC) and prefrontal cortical areas exerting top-down regulation on the thalamus and the amygdala in turn. In addition, the basal ganglia are activated through multiple pathways including the thalamus, the amygdala and cortical areas (Borsook et al., 2010). While areas that are involved in basic sensory pain processing, such as the PIC, are predominantly activated contralateral to the pain stimulus, higher-level processing areas implicated in pain interpretation including the AIC are activated in a bilateral fashion (Brooks et al., 2002).

Ongoing nociceptive input from injuries leads to a hyperexcitability of the nervous system, in a process that resembles long-term potentiation called central sensitization (Drdla and Sandkuhler, 2008; Woolf, 2011), in addition to a decrease of tonic inhibition (Moore et al., 2002; Keller et al., 2007). This hyperalgesia has the purpose of facilitating the healing processes of the injured tissue. However, central sensitization can persist after tissue healing leading to chronic hyperalgesia and even pain perception in the absence of painful stimuli (Voscopoulos and Lema, 2010; Woolf, 2011). Furthermore, pathological changes in the descending modulatory pathways might also contribute to the emergence of chronic pain (Porreca et al., 2002; Ossipov et al., 2010).

Functional brain imaging showed abnormal activation in the rostral ACC and the frontal cortex in certain chronic pain populations (Baliki et al., 2006; Berman et al., 2008; Jensen et al., 2009; Burgmer et al., 2010). Additionally, chronic pain patients show altered functional connectivity of the prefrontal cortex (PFC) and the insula with the default mode network (Napadow et al., 2010; Baliki et al., 2011). Similarly, structural imaging revealed gray matter reductions within the PFC, the ACC and the IC (Bushnell et al., 2013). On a molecular level, chronic pain patients seem to show altered endogenous release for the glutamatergic and GABAergic system as well as a decrease in receptor binding of the opioidergic system in these areas (Bushnell et al., 2013). These anatomical and molecular changes might not only alter pain regulation, but also affect decision making (Grace et al., 1999; Leavitt and Katz, 2006; Munguia-Izquierdo and Legaz-Arrese, 2007).

Some studies also suggest that these changes can be partly reversed, for example, in cases where there is an underlying painful condition that can be removed after years (Gwilym et al., 2010; Seminowicz et al., 2011). Moreover, the pain modulation system consisting of the PFC, ACC, and AIC was shown to be modulated by cognitive measures such as meditation or cognitive behavior therapy (Grant et al., 2011; Gard et al., 2012; Jensen et al., 2012). Thus, it seems useful and feasible to regulate these areas using rt-fMRI neurofeedback. Before looking into possible neurofeedback effects for chronic pain patients, we aim to optimize target ROI selection for pain neurofeedback in healthy subjects during pain stimulation as a first step. Future studies are needed to make sure that these target ROIs can be regulated in chronic pain patients as well.

The ACC and the AIC seem to be particularly important in perceiving pain intensity (Favilla et al., 2014). Therefore, these two regions of the medial pain system (Treede et al., 1999) were considered the most promising rt-fMRI target regions for cortical pain processing. The ACC was also the subject of a recent rt-fMRI neurofeedback study testing feasibility of pain regulation for the rostral ACC and PIC (Rance et al., 2014). They postulated that sensory pain aspects might be more related to PIC activation while affective aspects are more related to ACC activation. In this context, it is interesting to investigate how the AIC—implicated in another aspect of pain, namely cognitive control processes—can be regulated.

The ACC has been associated to several functions relevant to pain processing including saliency (Seeley et al., 2007; Iannetti and Mouraux, 2010), attention (Bush et al., 2000; Weissman et al., 2005), and emotion (Bush et al., 2000; Shackman et al., 2011). It is furthermore linked to affective processing of painful stimuli (Vogt et al., 1996; Rainville et al., 1997). Studies already showed that it is possible to target the ACC in smokers (Canterberry et al., 2013; Hartwell et al., 2013; Li et al., 2013) and chronic pain patients as well as healthy participants during pain perception (Decharms et al., 2005). In the latter study, regulation of the ACC activation using rt-fMRI neurofeedback even resulted in a decrease of pain intensity ratings. Other behavioral interventions that have been shown to modulate ACC activation include hypnosis (Rainville et al., 1997; Faymonville et al., 2000), modulation of pain expectation (Sawamoto et al., 2000; Bingel et al., 2011), and distraction (Bantick et al., 2002; Valet et al., 2004).

The IC can be divided into the anterior and the PIC that serve distinct functions in pain processing. The PIC seems to be involved in basic pain and touch sensation (Greenspan and Winfield, 1992), receiving direct spinothalamic input (Garcia-Larrea, 2012). Lesions in this area lead to pain and temperature deficits (Greenspan et al., 1999; Birklein et al., 2005). In contrast, AIC lesions usually do not seem to have a direct impact on pain perception *per se* (Greenspan et al., 1999). The AIC is implicated in a wide variety of functions, including visceral sensation and an integrative role in perception-action coupling possibly by mediating heightened alertness to prepare for action (Sterzer and Kleinschmidt, 2010). It seems to be engaged in affective-motivational processes of pain perception as a disconnection of the AIC from the PIC leads to a decrease of emotional pain reaction while nociceptive recognition remains intact (Berthier et al., 1988). Up-regulation of the AIC was shown to be possible (Caria et al., 2007; Veit et al., 2012) using recall of personal and affectively relevant events or focused attention on arising bodily sensations (Lawrence et al., 2013). It was shown that it is even possible to target subjects with clinical disorders such as schizophrenia (Ruiz et al., 2013) or depression (Linden et al., 2012). While these studies suggest that AIC regulation can be used to increase certain affective states and control, there is no specific data looking at the influence of the AIC down-regulation on pain perception.

In this work, we directly compared two possible target regions for rt-fMRI neurofeedback in pain, notably the AIC and the ACC, in order to determine the most efficient target region for future neurofeedback studies in pain processing.

## Materials and methods

### Participants

The local ethics committee in Mainz approved the study that adhered to the Declaration of Helsinki. Twenty-eight healthy subjects (mean age: 27.5 ± 2.3 years, 14 male, 14 female) gave written informed consent prior to participation. Participants were randomly split into two groups of *N* = 14 each, including seven male and seven female participants per group (group 1: 27.6 years ± 2.1, group 2: 27.4 ± 2.6 years). The first group received feedback from the left anterior insula (lAIC) as a target region, while the second group did so from the ACC. Exclusion criteria were defined by acute or chronic pain, pregnancy, severe neurological or internal disorders, intake of painkillers and contraindications for MR-measurements. Participants were paid for participation in the study.

### Real-time experiment

The experiment consisted of two stages. First, a functional localizer run with an ON-OFF block design of eight blocks alternating between continuous painful stimulation for 30 s and rest for 30 s each was performed to identify the individual target regions. The target region was chosen based on significant activation within the lAIC/ACC during the functional localizer. Thereafter, four identical neurofeedback runs were performed consisting of a block design of four rest and regulation blocks (30 s each) preceded by 15 s of initial rest before the first block. Online data analysis was performed using TurboBrainVoyager version 2.8 (Brain Innovation, Maastricht, The Netherlands).

The target region was chosen based on significant activation within the lAIC/ACC during the functional localizer (summarized in Supplementary Table 1). Regulation blocks included the same pain stimulation as during the localizer. During this period of the neurofeedback runs, subjects were asked to decrease the blood oxygen level dependent (BOLD) activation level in the target region, which was visualized to them by a yellow line. The background color of the yellow line indicated to either keep the yellow line constant (black = rest blocks, no heat pain) or to decrease the amplitude of the yellow line (blue = down-regulation, heat pain). Subjects could freely choose their mental strategy to reach this objective.

### Pain stimulation and rating

An MR compatible thermode (TSA 2001, Medoc Ltd, Ramat Yishai, Israel), placed at middle of the lower right volar forearm, was used for pain stimulation. This 30 × 30 mm Peltier device has a default temperature of 32°C. Before the start of the experiment the thermode temperature was adjusted for each participant to elicit a subjective pain intensity of 7 out of 10 on the numeric rating scale (NRS). The thermode temperature for pain stimulation remained constant throughout the experiment [Ramp rate: 4°C/s, mean ramp and fall time for AIC-group: 3.83 s (*SD* 0.26) and for ACC-group: 3.64 s (*SD* 0.32), mean plateau for AIC-Group: 22.35 s (*SD* 0.53) and for ACC-Group: 22.71 s (*SD* 0.64), mean temperature for AIC-Group: 47.08°C (*SD* 1.1) and for ACC-group: 46.42°C (*SD* 1.4)]. After each run pain ratings were obtained using a 11-point NRS ranging from 0 (not painful) to 10 (most painful).

### fMRI data acquisition

Imaging was performed on a 3T MRI Scanner (Siemens Tim Trio, Erlangen, Germany) using a 32-channel head-coil. For functional data acquisition an echo-planar imaging sequence (EPI, *TR* = 1500 ms, *TE* = 30 ms, matrix size 64 × 64, 24 slices, slice thickness 3 mm without gap) was utilized. Additionally, a high-resolution T1-weighted anatomical scan [magnetization prepared rapid gradient echo (MPRAGE), 1 mm isotropic] was acquired for later co-registration with the lower resolution EPI images.

### Statistical analysis between runs and groups

Statistical testing for differences between runs and groups [pain ratings, region of interest (ROI) activation, s-modes] was performed in MATLAB 2012b (The MathWorks, Inc., Natick, USA). First, parameters were tested for normality using D'Agostino K-squared test. As normality was rejected for all our parameters of interest (pain ratings, ROI beta values, s-mode values), we used the non-parametric Friedman test (comparison between all runs) and *post-hoc* Wilcoxon tests (comparison between groups, and comparison of two runs when the Friedman test showed significant results). Bonferroni correction was applied to correct for multiple comparisons in the s-mode analysis (i.e., the number of independent components).

### *Post-hoc* GLM activation analysis of the functional localizer

Off-line analysis was performed with SPM 8 (UCL, London, UK) and FSL 5.0 (FMRIB Analysis Group, University of Oxford, UK). Functional data was spatially realigned, co-registered to the anatomical data, normalized and smoothed (8 mm kernel) before group analysis on the basis of a general linear model (GLM) using the block design described under Section Real-Time Experiment. For the fMRI analysis, family-wise error (FWE) corrected values of *p* < 0.05 are considered significant.

### *Post-hoc* ROI activation analysis of the neurofeedback runs

GLM analysis for all four neurofeedback runs was performed analogous to the localizer run. As self-regulation was expected to increase with practice, we compared the first neurofeedback run with the subsequent runs in a ROI analysis for regions that were activated during the localizer run and known to be involved in pain processing, namely ACC, AIC, PIC. Based on our functional connectivity and ICA results in combination with its know implication in pain processing (Borsook et al., 2010), we included the caudate nucleus as an additional (a posteriori) ROI. Then, ROIs were defined as spheres with 1-cm diameter centered at the activation peaks within the relevant clusters from the group analysis of the functional localizer. This approach seemed more suitable than defining the ROIs on an individual level, as done for target ROI analysis, as not all subjects showed significant activation in all of the ROIs in the localizer run. Since regulation using rt-fMRI neurofeedback fails in some subjects, we restricted extensive *post-hoc* ROI analysis to those subjects who showed a decrease in activation in the target ROI; i.e., 9/14 for the AIC group and 8/14 subjects for the ACC group.

### *Post-hoc* fMRI connectivity analysis of the neurofeedback runs

Using FSL 5.0, functional connectivity was assessed with a seed-based approach testing for correlation with the seed's time course orthogonalized to the global signal and the GLM regressor of main effect. Seed regions were both rt-fMRI targets, ACC and lAIC, respectively. The resulting connectivity maps of each subject were fed into a 2nd level GLM analysis to obtain group results.

In addition, an independent component analysis (ICA) was carried out in FSL using multi-session multivariate exploratory linear optimized decomposition into independent components (MELODIC) tensor ICA. So-called s-modes (i.e., measures of activation strength for every component in each subject) were compared between groups.

## Results

### Effect of neurofeedback on pain ratings

**Figure 1:**
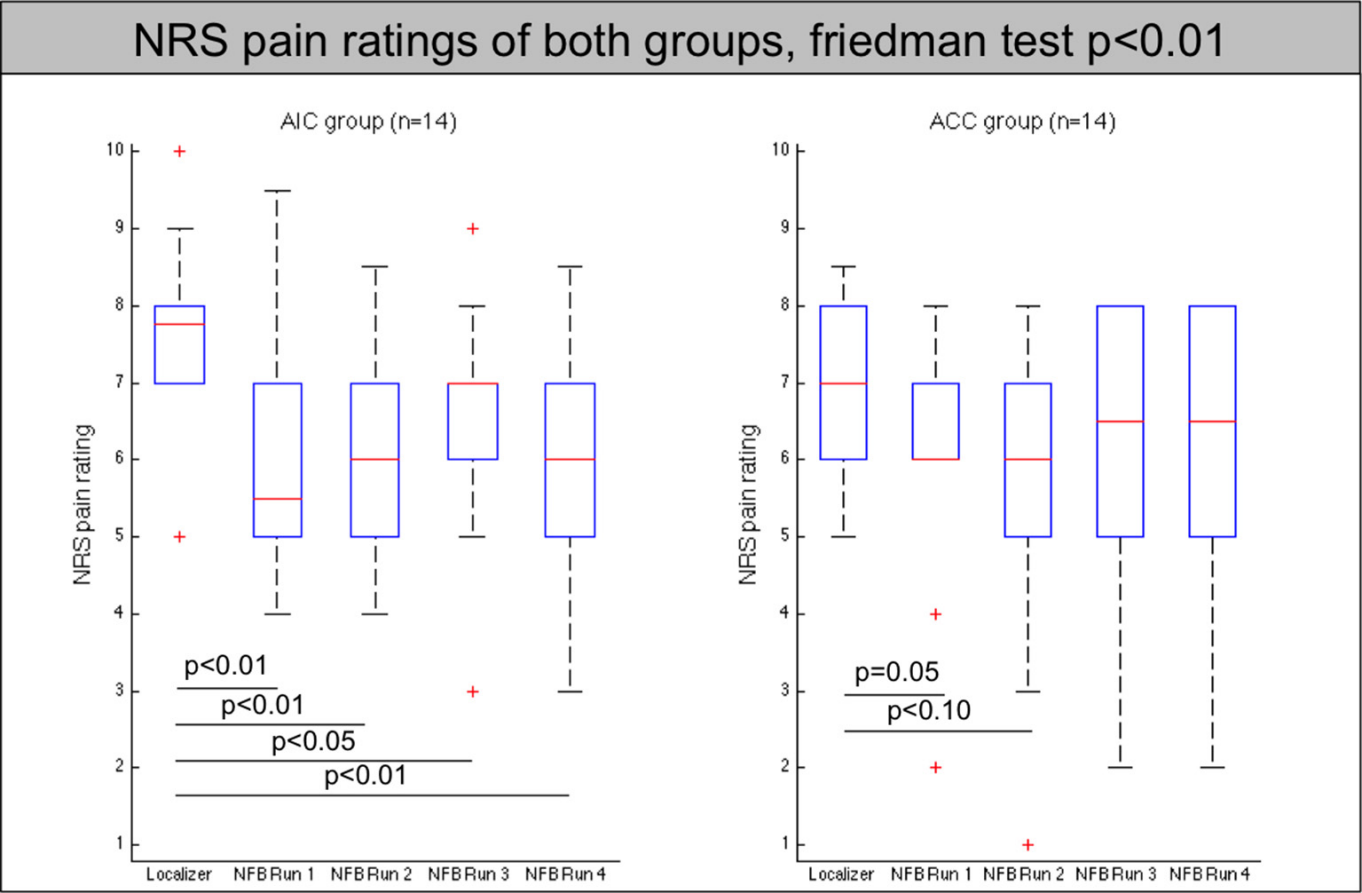
**Pain ratings of all participants (AIC-left, ACC-right) across localizer run and all neurofeedback runs**. The red line indicates the mean value, the box indicates 25%/75% confidence intervals and the whiskers indicate the most extreme points within 1.5 times of the box length.

Pain ratings were lower in the neurofeedback runs compared to the localizer run [non-parametric, p(AIC group) < 0.001; p(ACC) < 0.01] in both groups, but did not show any significant differences between neurofeedback runs (see Figure 1, Table 1). Pain ratings did not differ between regulators and non-regulators (*p* > 0.1).

**Table 1:** **Pain ratings on the numeric rating scale for all subjects**.

**Target ROI**	**Subject**	**Pain rating (0–10)**				
		**Localizer run**	**Run 1**	**Run 2**	**Run 3**	**Run 4**
AIC	1	8.0	4.0	4.0	6.0	5.0
	2	7.0	5.0	6.0	6.0	6.0
	3	8.0	6.0	5.0	7.0	7.0
	4	7.0	5.0	6.0	6.0	4.0
	5	5.0	5.0	7.0	7.0	7.0
	6	9.0	7.0	6.0	7.0	4.0
	7	8.0	8.0	8.0	7.0	7.0
	8	7.0	5.0	5.0	7.0	6.0
	9	7.0	6.0	6.0	5.0	5.0
	10	7.5	7.0	6.0	7.0	6.0
	11	10.0	9.5	7.5	7.5	7.5
	12	9.0	7.5	8.5	9.0	8.5
	13	8.0	5.0	6.0	8.0	8.0
	14	7.0	5.0	4.0	3.0	3.0
ACC	15	6.0	6.0	7.0	8.0	8.0
	16	7.0	6.0	6.0	4.0	6.0
	17	8.0	8.0	8.0	8.0	8.0
	18	6.0	6.0	6.0	6.0	5.0
	19	8.0	7.0	7.0	8.0	8.0
	20	7.0	6.0	6.0	8.0	7.0
	21	8.5	6.0	6.0	6.0	6.0
	22	7.0	6.0	5.0	6.0	5.0
	23	7.0	6.0	5.0	5.0	6.0
	24	5.0	4.0	3.0	4.0	4.0
	25	8.0	7.0	7.0	7.0	7.0
	26	8.0	8.0	8.0	8.0	8.0
	27	8.0	6.0	7.0	8.0	7.0
	28	5.0	2.0	1.0	2.0	2.0

Neither pain ratings of the regulators nor the non-regulators changed significantly between neurofeedback runs.

### Functional localizer

**Figure 2:**
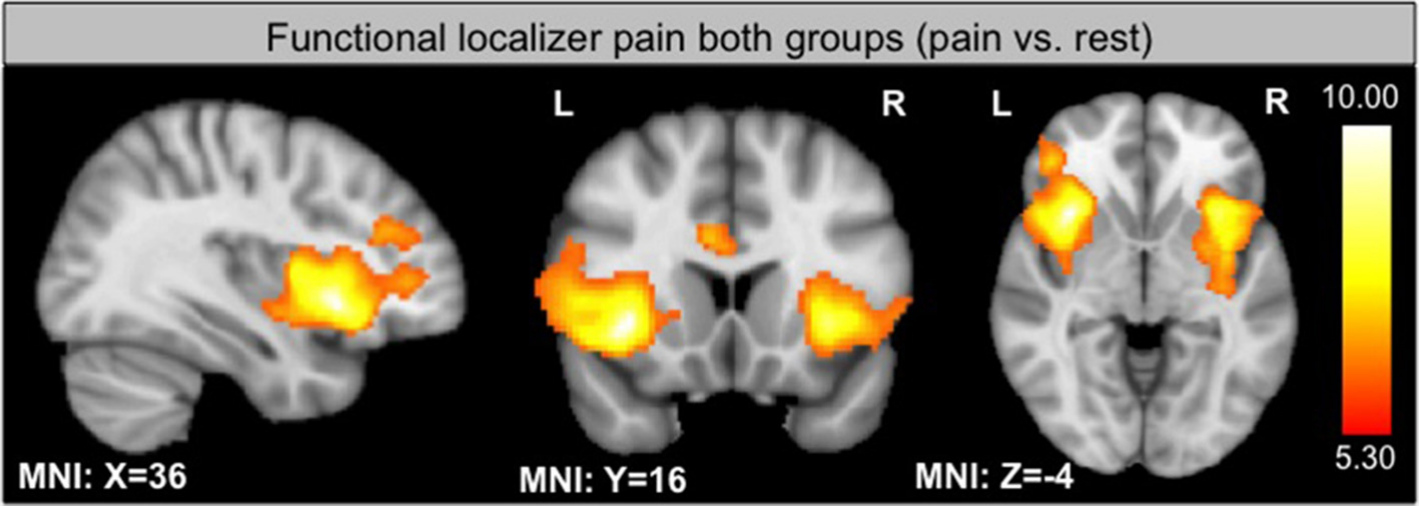
**GLM contrast localizer run: pain vs. rest in both groups threshold at *p* < 0.05 FWE**.

As expected, the functional localizer revealed significant activation within the insula, PFC and the ACC, all regions involved in pain processing (see Figure 2). Activation of the target region in each subject enabled the individual region of interest placement (see Supplementary Figure 1).

### Neurofeedback runs

#### Seed-based connectivity of the left AIC and the ACC

**Figure 3:**
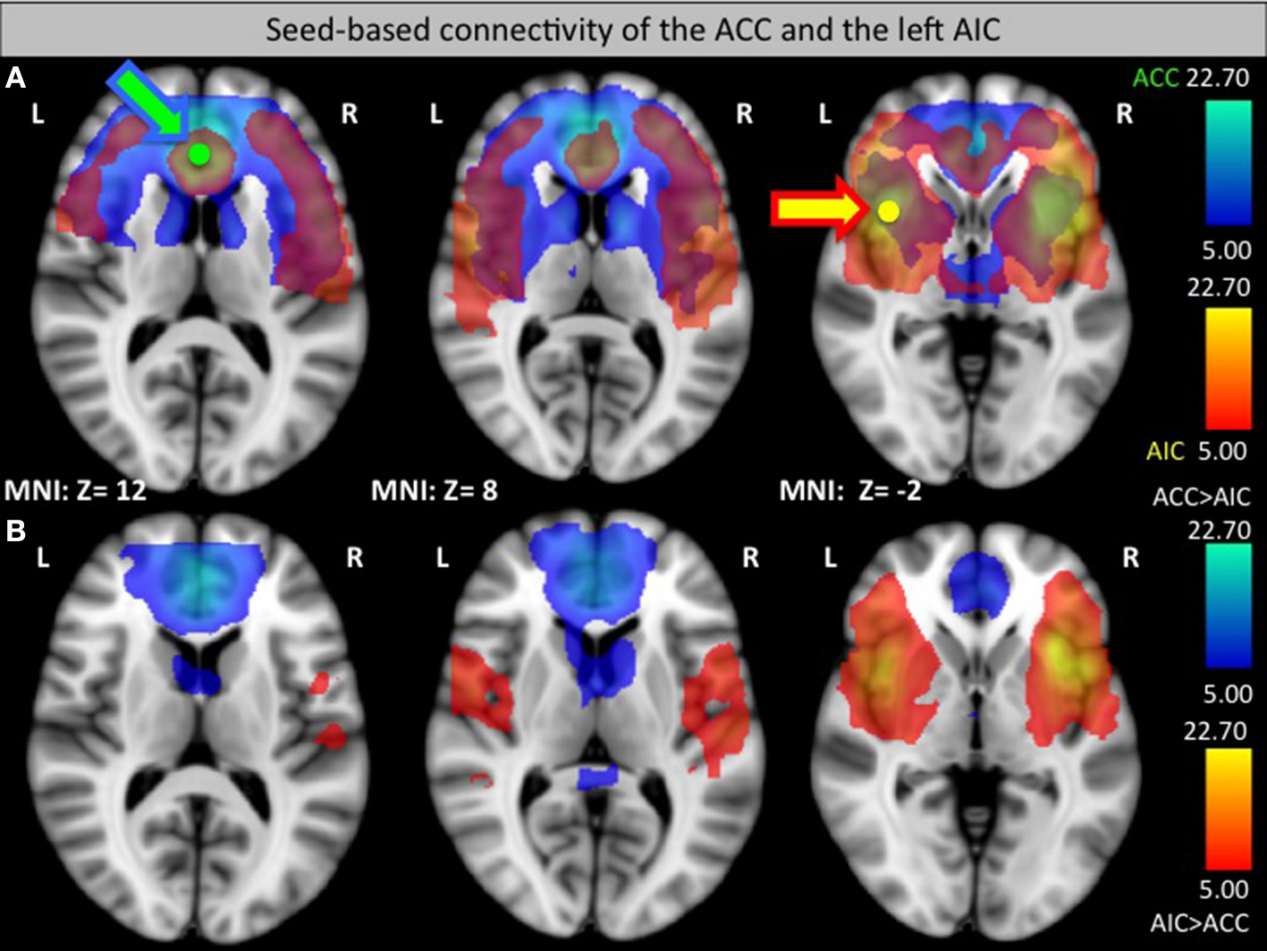
**Seed-based functional connectivity of the ACC (green, connectivity blue, A) and left AIC (yellow, connectivity orange, A). (B)** Areas that had a significantly greater connection to the ACC than to the AIC (blue) or a significantly greater connection to the AIC than to the ACC (orange) in a direct comparison. Arrows indicate the target seed location.

Seed-based analysis at the group level showed the functional connectivity of the ACC and the AIC to other regions of the pain network (see Figure 3A). The analysis confirmed that ACC and the AIC are strongly interconnected as well as showing connections to prefrontal areas. Interestingly, the ACC has high functional connectivity with the caudate nucleus that did not show up in the AIC connectivity map while the AIC group has an increased connectivity with the ventrolateral PFC (see Figure 3B).

#### Effect of training (runs over time)

**Figure 4:**
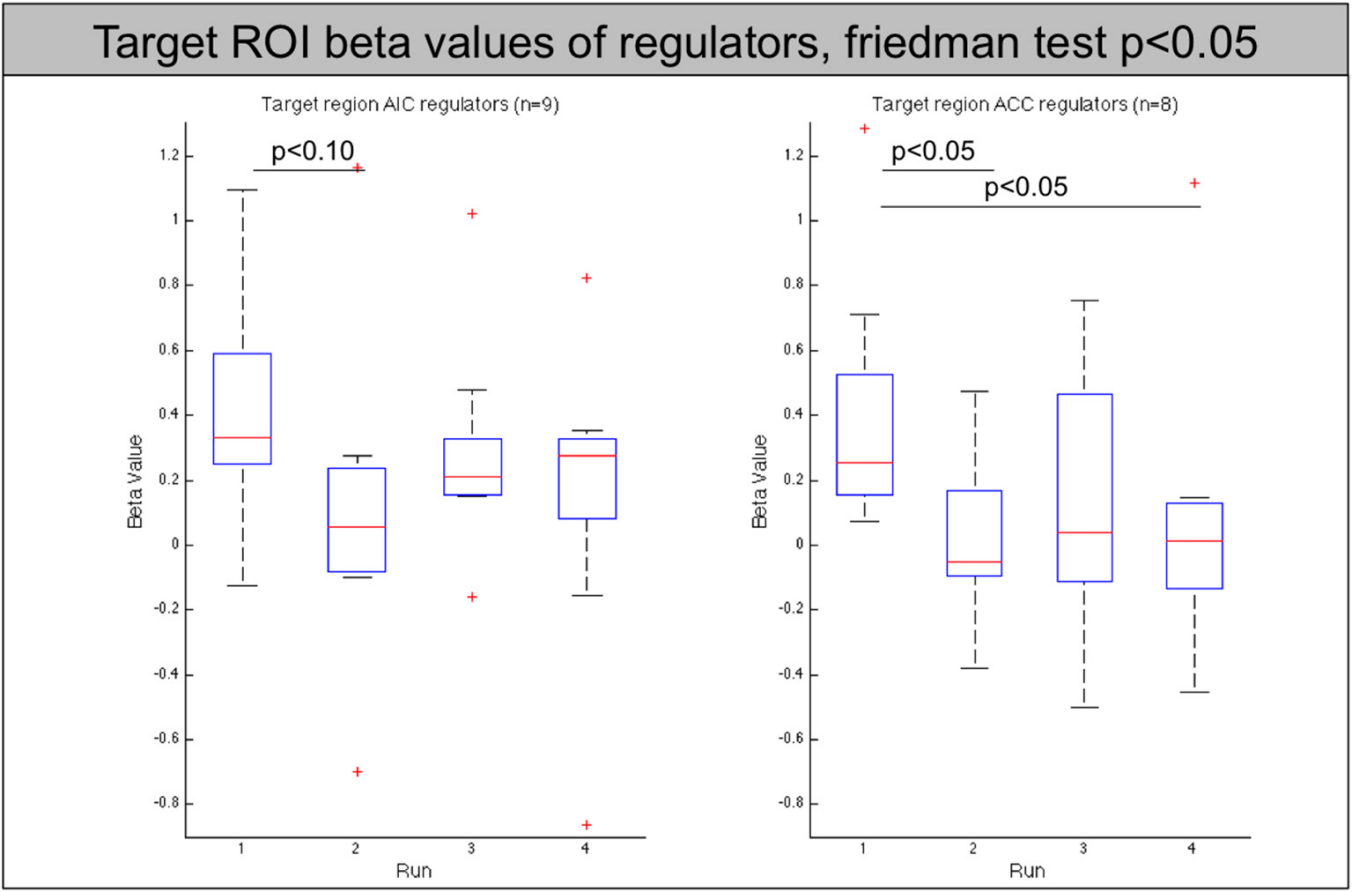
**Beta values of the target region (left AIC/ACC) for all regulators across neurofeedback runs (AIC-left, ACC-right)**. The red line indicates the mean value, the box indicates 25%/75% confidence intervals and the whiskers indicate the most extreme points within 1.5 times of the box length.

To assess a possible improvement in self-regulation over time, we looked for a decrease in activation in the later runs compared to the first run. To that aim, we first analyzed the activation within each individual target ROI (see Table 2 and Figure 4). Data from subjects that showed a successful down-regulation (i.e., decrease of target region's activation level from the first to the average of the following runs) were used for a more extensive ROI analysis including the main brain areas involved in pain regulation (see Table 3).

**Table 2:** **Beta values of the target ROI for all subjects, classification criteria (beta value decrease from run 1 to the average of run 2–4), and classification label (+, regulator; −, non-regulator)**.

**Target ROI**	**Subject**	**Beta value**				**Beta value decrease**	**Regulator**
		**Run 1**	**Run 2**	**Run 3**	**Run 4**		
AIC	1	0.156	0.097	0.400	0.285	−0.315	−
	2	0.332	−0.077	0.212	0.277	0.584	+
	3	0.436	0.274	0.227	0.162	0.643	+
	4	−0.125	−0.698	0.183	−0.862	1.002	+
	5	0.204	0.006	0.462	0.753	−0.608	+
	6	−0.481	−0.367	0.252	−0.290	−1.039	−
	7	0.325	0.224	0.157	0.201	0.395	+
	8	0.446	−0.099	0.274	0.319	0.843	+
	9	1.093	1.163	0.479	0.822	0.816	−
	10	0.268	0.056	−0.161	0.354	0.556	+
	11	1.026	−0.059	1.022	0.313	1.803	+
	12	0.201	0.104	0.149	−0.155	0.506	+
	13	0.257	0.413	0.825	0.353	−0.819	−
	14	−0.008	0.044	0.246	0.422	−0.737	−
ACC	15	0.1934	−0.0628	−0.1711	0.1088	0.705	+
	16	−0.147	−0.101	0.146	−0.162	−0.325	+
	17	0.072	−0.119	−0.502	−0.182	1.020	+
	18	−0.127	0.072	0.060	0.061	−0.575	−
	19	0.341	−0.379	1.795	−0.455	0.063	+
	20	0.281	0.221	0.178	0.013	0.432	−
	21	0.240	0.847	0.309	0.324	−0.760	−
	22	0.117	−0.071	0.097	0.146	0.179	+
	23	−0.008	0.429	−0.610	0.254	−0.097	+
	24	0.450	0.719	0.874	0.943	−1.186	−
	25	0.223	−0.046	−0.051	0.012	0.754	+
	26	0.713	0.476	−0.020	−0.085	1.766	+
	27	0.153	0.248	0.324	0.309	−0.424	−
	28	1.284	0.116	0.754	1.116	1.866	−

**Table 3:**
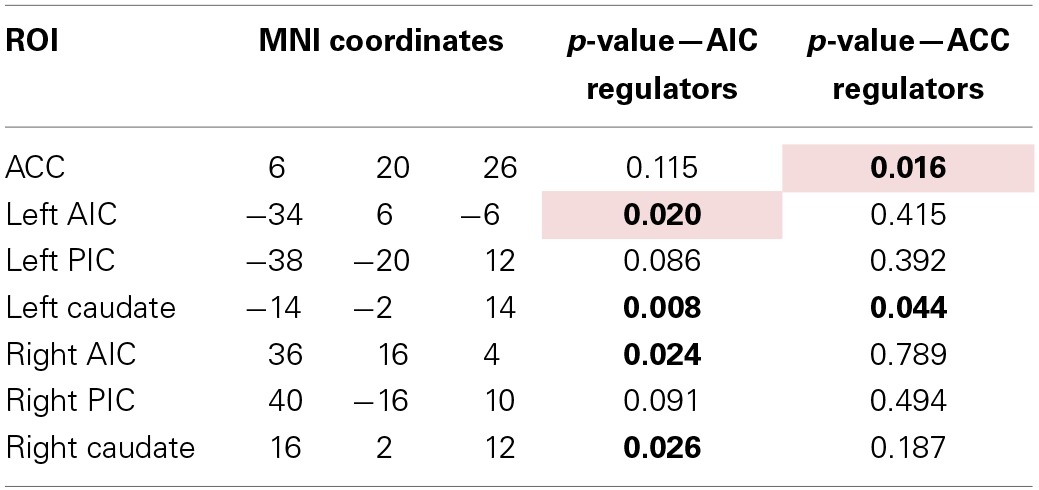
**Overview of ROIs with their location and *p*-value of Friedman test for change in Beta-value across neurofeedback runs (AIC: *n* = 9, ACC: *n* = 8)**.

*Bold numbers indicate significant results (*p* < 0.05), values for the corresponding target area are highlighted red*.

**Figure 5:**
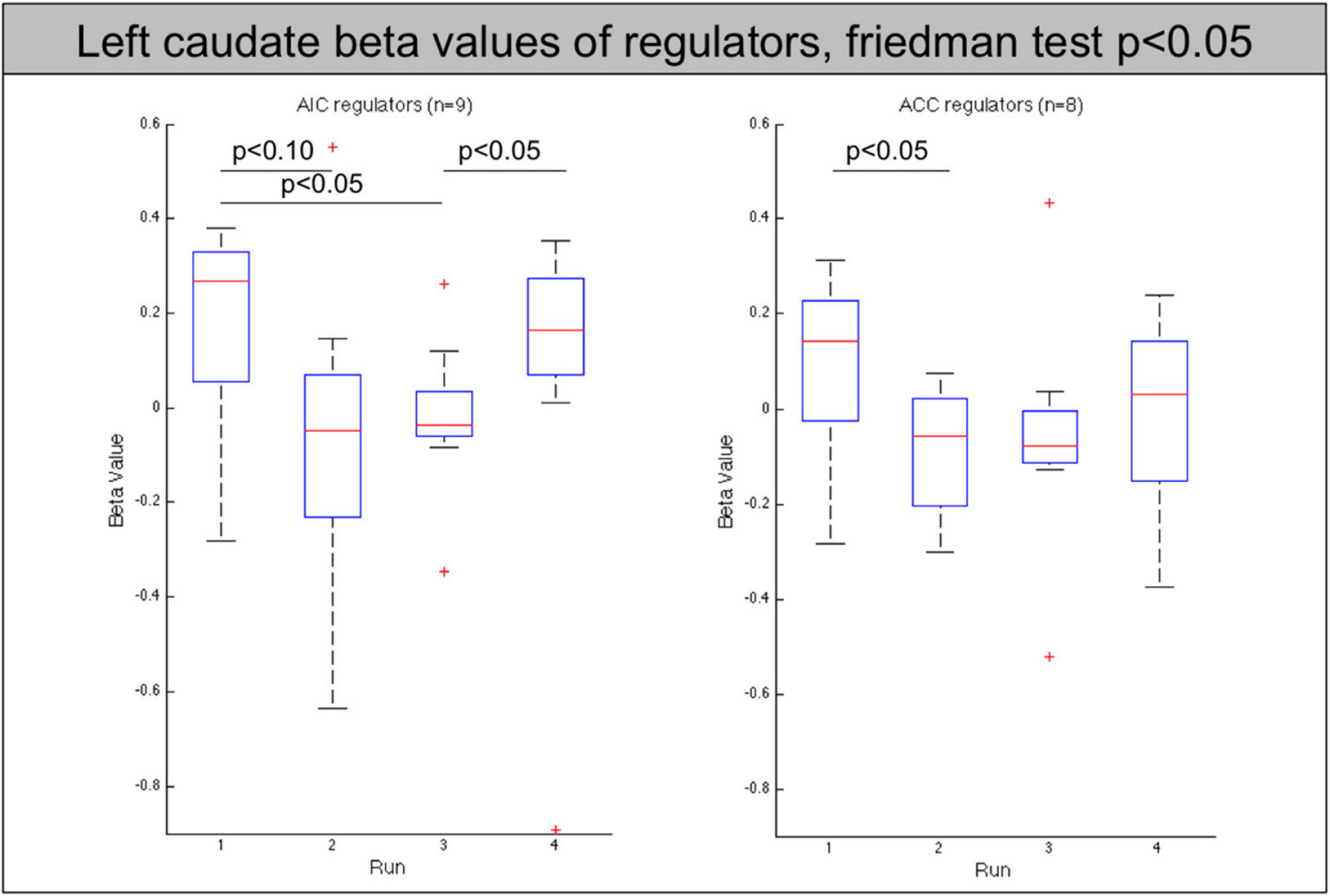
**Beta values of the left caudate nucleus for all regulators across neurofeedback runs (AIC-left, ACC-right)**. The red line indicates the mean value, the box indicates 25%/75% confidence intervals and the whiskers indicate the most extreme points within 1.5 times of the box length.

The analysis of these regions showed that the decrease of the left AIC in the AIC group is accompanied by a similar significant decrease in the contralateral anterior insula (*p* < 0.05). In addition, both groups show a significant decrease of the caudate nucleus with the effect being more pronounced in the AIC group (*p* < 0.01, ACC: *p* < 0.05, see Figure 5).

#### Independent component analysis

**Figure 6:**
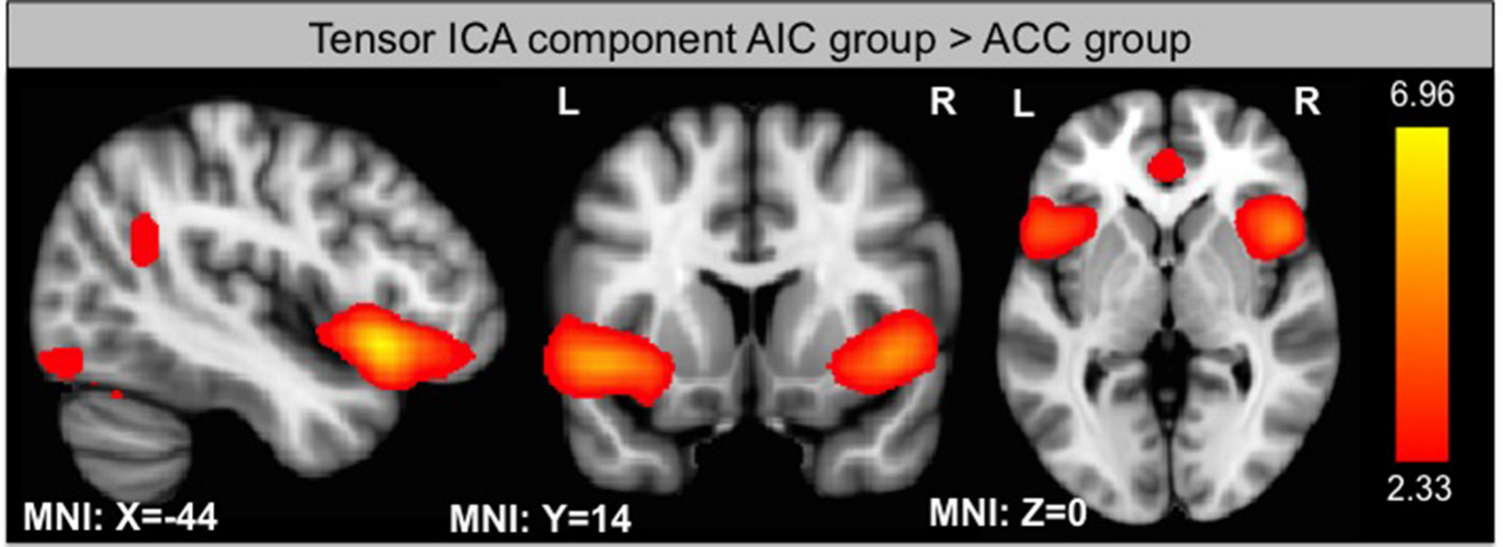
**Component from ICA that shows significantly different s-mode values between group**.

**Figure 7:**
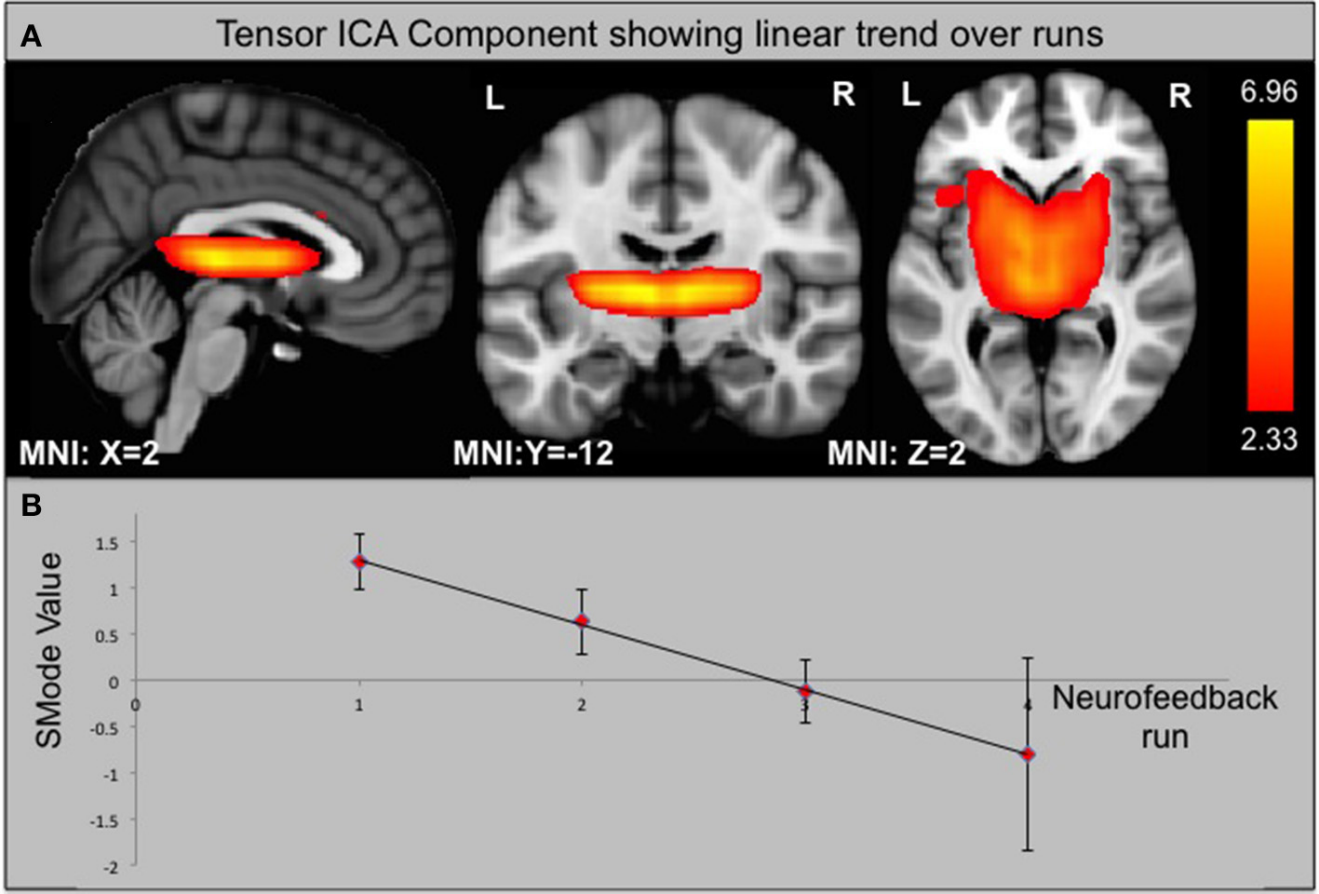
**Component from ICA that shows a significant linear trend over neurofeedback runs in the ACC group. (A)** Component identified by ICA. **(B)** SMode Values of the component shown in **(A)** over runs. Error bars indicate the standard error of the mean (SEM).

Using ICA, we identified 33 components of which one is significantly different between groups according to its s-mode (*p* < 0.05, corrected for multiple comparison). This component includes AIC, ACC, and small portions of the occipital and parietal lobes (see Figure 6). In addition, we looked for components that exhibit a linear trend over runs and identified one component with slope significantly different from zero for the ACC group (*p* < 0.05, corrected for multiple comparison). This component includes thalamus and parts of the basal ganglia (see Figure 7).

## Discussion

In the current investigation, we compared the effectiveness in pain regulation using real-time fMRI neurofeedback from two different target regions, notably ACC and AIC. At the behavioral level, both for ACC and AIC feedback, the neurofeedback runs showed a decrease in pain perception with respect to the identical pain stimulation in the localizer runs. However, there was no significant behavioral difference in the direct comparison between ACC and AIC and between runs. Despite the absence of behavioral differences between runs, we found effects in neuroimaging for the two target regions. This observation is in line with the known higher sensitivity of neuroimaging, as compared to behavioral measures, in functional MRI studies investigating subtle effects (Weiskopf et al., 2003; Haller et al., 2005, 2013; Johnston et al., 2011).

At the neuroimaging level, AIC and ACC regulation led to a significant down-regulation of parts of the pain network with practice, notably the caudate nucleus for successful regulators.

Functional-connectivity analyses further demonstrated that both target regions are functionally well connected to other parts of the pain network. Therefore, based on this neuroimaging evidence, we found that both AIC and ACC influence the pain network in a similar fashion through the caudate nucleus.

### ACC regulation during pain

Contrary to two previous studies about rt-fMRI ACC regulation of pain processing (Decharms et al., 2005; Rance et al., 2014), we did not find a significant down-regulation effect of ACC regulation over runs within the ACC for all subjects. This might be due to the different experimental paradigm that compared down-regulation vs. no regulation in our setting, while deCharms et al. compared up- vs. down-regulation. Considering that down-regulation might be harder to obtain than up-regulation, as it is easier to explicitly focus on acute pain than to find a strategy to decrease pain, the effect of down-regulation might be smaller. In addition, this particular finding could not be replicated by deCharms et al. in a later follow-up study; as publicly stated at the rt-fMRI conference in Zurich, 2012 (Decharms, 2012). One factor that possibly complicates ACC regulation is that the adjacent anterior mid-cingulate cortex (aMCC) is also thought to be involved in rt-fMRI neurofeedback regulation (Lee et al., 2012), inducing activation during the regulation and thus making it harder to detect the deactivation in nearby ACC, and possibly also confounding the participants' feedback signal itself to some extent. This possible confound is less strong in the recent study of Rance et al. as they used a more rostral part of the ACC leading to a significant down-regulation of this ROI. Therefore, future studies should preferably use a more rostral part of the ACC.

Nevertheless, ACC rt-fMRI neurofeedback did induce a down-regulation of the ACC in a large group of subjects (8/14) as well as a significant change within the caudate nucleus, a brain region involved in planning of goal directed actions (Grahn et al., 2008) and affective processing of pain (Borsook et al., 2010). This part of the basal ganglia is anatomically closely connected to the ACC, with functional relevance, for example, in pain avoidance behavior in monkeys (Koyama et al., 2000). Similarly, previous studies found caudate nucleus involvement when participants suppressed pain (Freund et al., 2009; Wunderlich et al., 2011). Thus, the caudate nucleus, regulated via the ACC, seems to be important in deliberate pain control. This result is supported by the seed-based functional connectivity analysis showing a strong ACC—caudate nucleus interaction and the ICA analysis that revealed a specific component including the caudate nucleus and thalamus that showed significantly decreasing s-modes as a function of runs. These results also indicate that the caudate, the thalamus or a combination of these regions could be considered as suitable targets for future pain real-time neurofeedback studies.

### AIC regulation during pain

Similar to the ACC group, AIC down-regulation was not significant when looking at all subjects. This difficulty in AIC regulation might be explained by competing processes within the AIC. On one hand, the AIC was selected as the target for down-regulation as it is a core component of the network involved in pain processing (Apkarian et al., 2005). On the other hand it is likely to be activated in neurofeedback regulation processes (Haller et al., 2010). In addition, the AIC is involved in many other cognitive processes such as saliency detection (Cauda et al., 2012) and emotion regulation and representation (Singer et al., 2004; Eippert et al., 2007). Due to the regulation procedure, saliency of the visual display (focus on the line and the lower part of the “scale”) as well as saliency of the pain stimulus (less focus on pain) could be modulated. In addition, the feedback could induce emotions such as frustration or contentment, thus possibly increasing insula activation, thereby counteracting insula down-regulation. This might also explain why all previous studies only reported reliable up-regulation while voluntary down-regulation of the AIC by rt-fMRI neurofeedback was less successful (Veit et al., 2012). The possible interaction of cognitive and emotional processes within the AIC was also underlined by an fMRI study showing increased reaction times and error rates for cognitively demanding tasks during presentation of painful compared to non-painful pictures (Gu et al., 2013).

However, 9 out of 14 subjects showed a trend to down-regulation of the AIC. In these subjects the ROI analysis also showed a down-regulation of the contralateral AIC. This corresponding contralateral change could be expected, given the bilateral processing of higher-level pain functions and the high connectivity between the left and right AIC as confirmed in the functional connectivity analysis. Additionally, the left and right caudate nucleus showed a down-regulation when comparing the first and later feedback runs. The fact that in both groups successful target region regulation is accompanied by a decrease in caudate nucleus activation underlines its importance in pain regulation.

### Differences in the functional connectivity and ICA between groups

Functional connectivity analysis revealed that the ACC shows a stronger functional connectivity to the caudate nucleus while the AIC is more heavily connected to the ventrolateral PFC. These differences might reflect different pathways of pain regulation. While the ACC might directly influence caudate nucleus activity, the AIC has a stronger connection to higher-level processing via the PFC that in turn might regulate caudate activity.

ICA revealed one functional connectivity ICA component involving the ACC and the AIC that showed significantly lower s-mode values (a measure of effect size) in the ACC group in comparison to the AIC group. This implies that AIC and ACC activity overall was higher in the AIC group. One possible explanation might be that AIC regulation is harder to obtain in the beginning due to competing processes within this brain region. This might lead to an increase in pain processing within the AIC and ACC that is compensated at a later phase when subjects learned down-regulation.

### Effect of rt-fMRI on pain ratings

In addition to our main goal of comparing two targets for rt-fMRI neurofeedback, we also looked at the pain rating as a function of runs. The finding that pain ratings decreased in neurofeedback runs compared to the localizer run suggests that ACC and AIC down-regulation by means of rt-fMRI neurofeedback decreases pain perception. Two contradictory factors potentially confound the interpretation of decrease in pain perception. Habituation might reduce, while sensitization might increase subjective pain perception despite identical physical stimulation. The observed result of decreased pain ratings in feedback as compared to localizer runs would not be expected from a regular pain study as short-term repeated pain stimulation in general causes sensitization rather than habituation (Drdla and Sandkuhler, 2008; Breimhorst et al., 2012). The same trend was seen in another recent pain real-time neurofeedback study (Rance et al., 2014) where slightly higher pain intensity was applied and pain unpleasantness ratings were compared for the last against the first run, indicating a pain sensitization over run. However, we cannot exclude the possibility that the placebo effect, caused by the neurofeedback intervention, might have confounded pain ratings during neurofeedback runs. Pain perception is known to vary depending on the context (Rhudy and Meagher, 2000; Iannetti et al., 2008; Wang and Mitchell, 2011), therefore, making it hard to distinguish the factors that contribute to the pain reduction between localizer run and feedback runs. The fact that subjects were directing attention toward a cognitively demanding task itself could decrease pain perception as shown in a study working with different distraction tasks (Verhoeven et al., 2011). Both effects might be particularly high in the first neurofeedback runs when the task is new and subjects exert more effort than later on, thus possibly counteracting the desired effect of increasing regulation. The difference between localizer and neurofeedback pain rating in the AIC group can also be explained by competing processes within the ROI and the effect of cognitively highly demanding task engagement. These confounding effects might be similar in size to the effects of rt-fMRI, which are expected to be rather small, considering that pain perception has been experienced for years while cognitive modulation of pain has been practiced for minutes only. Some other neuroimaging studies already showed a similar phenomenon: significant neuroimaging effects were not accompanied by corresponding behavioral changes (Weiskopf et al., 2003; Haller et al., 2005, 2009, 2013, 2014; Johnston et al., 2011). This might indicate that objective fMRI data are more sensitive to small-scale changes within a rather small group than subjective behavioral measures. Therefore, it is not surprising that the decreased caudate activity over runs in the AIC and ACC group did not directly lead to a significant decrease in pain rating between feedback runs.

### Strength and limitations

The current investigation is a comparison of two possible target regions for rt-fMRI neurofeedback in pain. It clearly indicates that the AIC and the ACC could serve as a pain neurofeedback target in future studies. The following limitations should however be taken into account when interpreting the current results. First, this study did not aim at assessing the absolute behavioral effect of neurofeedback on pain ratings. Thus, further studies including sham feedback as well as modified pain stimulation are needed to separate specific effects of rt-fMRI neurofeedback from habituation/sensitization over time. Additionally, these studies should aim to compare neurofeedback to a sham method with a similar cognitive load, as a high cognitive load could influence pain ratings as well (Verhoeven et al., 2011). Second, as in previous real-time fMRI studies (Decharms et al., 2005; Bray et al., 2007; Scharnowski et al., 2012; Robineau et al., 2014) not all subjects learned to down regulate the target area. Future studies should aim at identifying the parameters that lead to successful rtfMRI neurofeedback regulation in order to maximize the number of subjects that succeed. Another limitation lies in the use of a GLM on the basis of a box-type function convolved with the hemodynamic response function. Due to this hypothesis about the shape of the response, differently shaped responses such as a decrease in BOLD response after a certain period of pain stimulation, as it has been reported for the thalamus (Tran et al., 2010), would lead to underestimated statistical values.

The ACC and the AIC were judged as the most suitable neurofeedback targets based on literature (see Introduction). Based on our results the caudate nucleus and the thalamus or measures of the connectivity between the ACC and the caudate nucleus (e.g., intrinsic connectivity contrast degree) might be an additional target for future rt-fMRI neurofeedback studies in the domain of pain. As a next step, the potential long-term effects of neurofeedback training on pain perception should be assessed using the AIC, the ACC, thalamus or caudate nucleus as ROI in healthy subjects and as a next step also in chronic pain patients. Due to the possible involvement of the aMCC in neurofeedback regulation processes, the target area should be sufficiently separated from the aMCC. These future studies could be another important step toward a possible supplemental pain therapy to reduce the impact of pain on patients' life.

### Conflict of interest statement

The authors declare that the research was conducted in the absence of any commercial or financial relationships that could be construed as a potential conflict of interest.

## Abbreviations

ACC: anterior cingulate cortex
AIC: anterior insular cortex
aMCC: anterior mid-cingulate cortex
ANOVA: analysis of variance
BOLD: blood oxygenation level dependent
fMRI: functional magnetic resonance imaging
GLM: general linear model
ICA: independent component analysis
MELODIC: multivariate exploratory linear optimized decomposition into independent components
MPRAGE: magnetization prepared rapid gradient echo
NRS: numeric rating scale
PIC: posterior insular cortex
PFC: prefrontal cortex
ROI: region of interest
rt-fMRI: real-time fMRI
SEM: standard error of the mean.
