# Supplementary material for: Comparison of anterior cingulate vs. insular cortex as targets for real-time fMRI regulation during pain stimulation
Source: Front Behav Neurosci. 2014 Oct 9;8:350. doi: 10.3389/fnbeh.2014.00350 (PMC4191436; doi:10.3389/fnbeh.2014.00350)
Supplement: Supplementary file 1 [file Table1.DOCX]

Supplementary material

|  |  | ROI -center of gravity (MNI) | | |  |  |
| --- | --- | --- | --- | --- | --- | --- |
| Target ROI | Subject | X | Y | Z | Volume of ROI [mm^3^] | Number of voxels |
| AIC | 1 | -33.02 | 4.81 | 1.84 | 3267 | 121 |
|  | 2 | -34.73 | 5.14 | 8.75 | 2565 | 95 |
|  | 3 | -37.61 | 12.33 | 8.19 | 2943 | 109 |
|  | 4 | -40.37 | 8.09 | 7.40 | 4131 | 153 |
|  | 5 | -33.85 | 4.56 | 13.95 | 3996 | 148 |
|  | 6 | -35.35 | -2.63 | 5.02 | 3348 | 124 |
|  | 7 | -35.96 | 1.82 | 14.44 | 4563 | 169 |
|  | 8 | -32.61 | 4.44 | 6.21 | 3213 | 119 |
|  | 9 | -44.40 | 8.34 | 1.45 | 3807 | 141 |
|  | 10 | -33.41 | 13.02 | 16.76 | 729 | 27 |
|  | 11 | -44.66 | 0.81 | -4.10 | 1620 | 60 |
|  | 12 | -29.73 | 12.96 | 2.65 | 2700 | 100 |
|  | 13 | -31.96 | 37.04 | 10.38 | 2646 | 98 |
|  | 14 | -38.58 | 14.73 | 16.47 | 1269 | 47 |
| ACC | 15 | -4.91 | -0.77 | 39.91 | 837 | 31 |
|  | 16 | -7.07 | -9.64 | 39.94 | 432 | 16 |
|  | 17 | 1.81 | 7.32 | 42.84 | 3159 | 117 |
|  | 18 | 1.27 | 14.56 | 38.34 | 918 | 34 |
|  | 19 | -0.81 | 18.52 | 25.33 | 1782 | 66 |
|  | 20 | 1.24 | 12.90 | 35.17 | 1755 | 65 |
|  | 21 | 2.53 | 11.99 | 30.02 | 675 | 25 |
|  | 22 | 11.48 | 17.19 | 32.31 | 324 | 12 |
|  | 23 | 6.47 | 15.75 | 44.17 | 1539 | 57 |
|  | 24 | 5.29 | 20.28 | 27.03 | 162 | 6 |
|  | 25 | 4.94 | 23.35 | 22.82 | 378 | 14 |
|  | 26 | -3.05 | 27.02 | 36.14 | 3510 | 130 |
|  | 27 | -5.32 | 0.50 | 45.29 | 1863 | 69 |
|  | 28 | 2.03 | 6.50 | 44.07 | 810 | 30 |

Supplementary table 1: Location and extent of target ROIs for all subjects.

Supplementary Figure Legends:

Supplementary Figure 1: Group average of location of the target ROIs for the AIC (left) and AIC (right) group.
