# Supplementary figures and images for: Comparison of anterior cingulate vs. insular cortex as targets for real-time fMRI regulation during pain stimulation
Source: Front Behav Neurosci. 2014 Oct 9;8:350. doi: 10.3389/fnbeh.2014.00350 (PMC4191436; doi:10.3389/fnbeh.2014.00350)

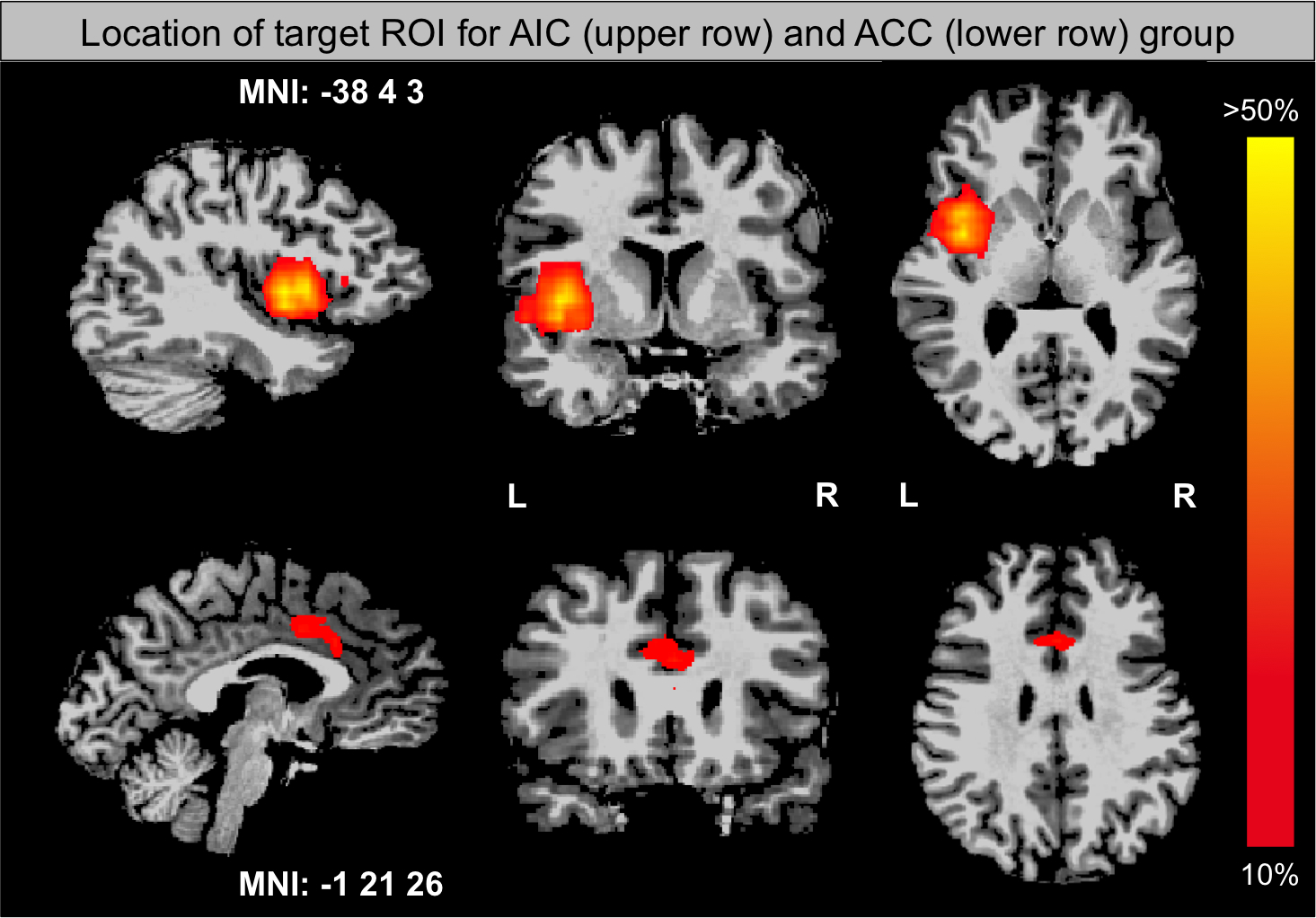

Supplement: Supplementary file 2 [file Image1.JPEG]
